# The role of autophagy in cardiovascular disease: Cross-interference of signaling pathways and underlying therapeutic targets

**DOI:** 10.3389/fcvm.2023.1088575

**Published:** 2023-03-29

**Authors:** Bing Jiang, Xuan Zhou, Tao Yang, Linlin Wang, Longfei Feng, Zheng Wang, Jin Xu, Weiyao Jing, Tao Wang, Haixiang Su, GuoWei Yang, Zheng Zhang

**Affiliations:** ^1^ Department of Integrated Chinese and Western Medicine, Gansu University of Traditional Chinese Medicine, Lanzhou, China; ^2^ Department of First Clinical Medicine, Gansu University of Traditional Chinese Medicine, Lanzhou, China; ^3^ Department of Basic Medicine, Gansu University of Traditional Chinese Medicine, Lanzhou, China; ^4^ Department of First Clinical Medicine, Lanzhou University, Lanzhou, China; ^5^ Department of Acupuncture-Moxibustion and Tuina, Gansu University of Traditional Chinese Medicine, Lanzhou, China; ^6^ Research Center for Translational Medicine, Gansu Province Academic Institute for Medical Research, Gansu Provincial Cancer Hospital, Lanzhou, China; ^7^ Center for Heart, First Hospital of Lanzhou University, Lanzhou, China

**Keywords:** autophagy, cardiovascular disease, autophagy-related gene, signaling pathway, crosstalk, potential target

## Abstract

Autophagy is a conserved lysosomal pathway for the degradation of cytoplasmic proteins and organelles, which realizes the metabolic needs of cells and the renewal of organelles. Autophagy-related genes (ATGs) are the main molecular mechanisms controlling autophagy, and their functions can coordinate the whole autophagic process. Autophagy can also play a role in cardiovascular disease through several key signaling pathways, including PI3K/Akt/mTOR, IGF/EGF, AMPK/mTOR, MAPKs, p53, Nrf2/p62, Wnt/β-catenin and NF-κB pathways. In this paper, we reviewed the signaling pathway of cross-interference between autophagy and cardiovascular diseases, and analyzed the development status of novel cardiovascular disease treatment by targeting the core molecular mechanism of autophagy as well as the critical signaling pathway. Induction or inhibition of autophagy through molecular mechanisms and signaling pathways can provide therapeutic benefits for patients. Meanwhile, we hope to provide a unique insight into cardiovascular treatment strategies by understanding the molecular mechanism and signaling pathway of crosstalk between autophagy and cardiovascular diseases.

## Introduction

**Figure 1:**
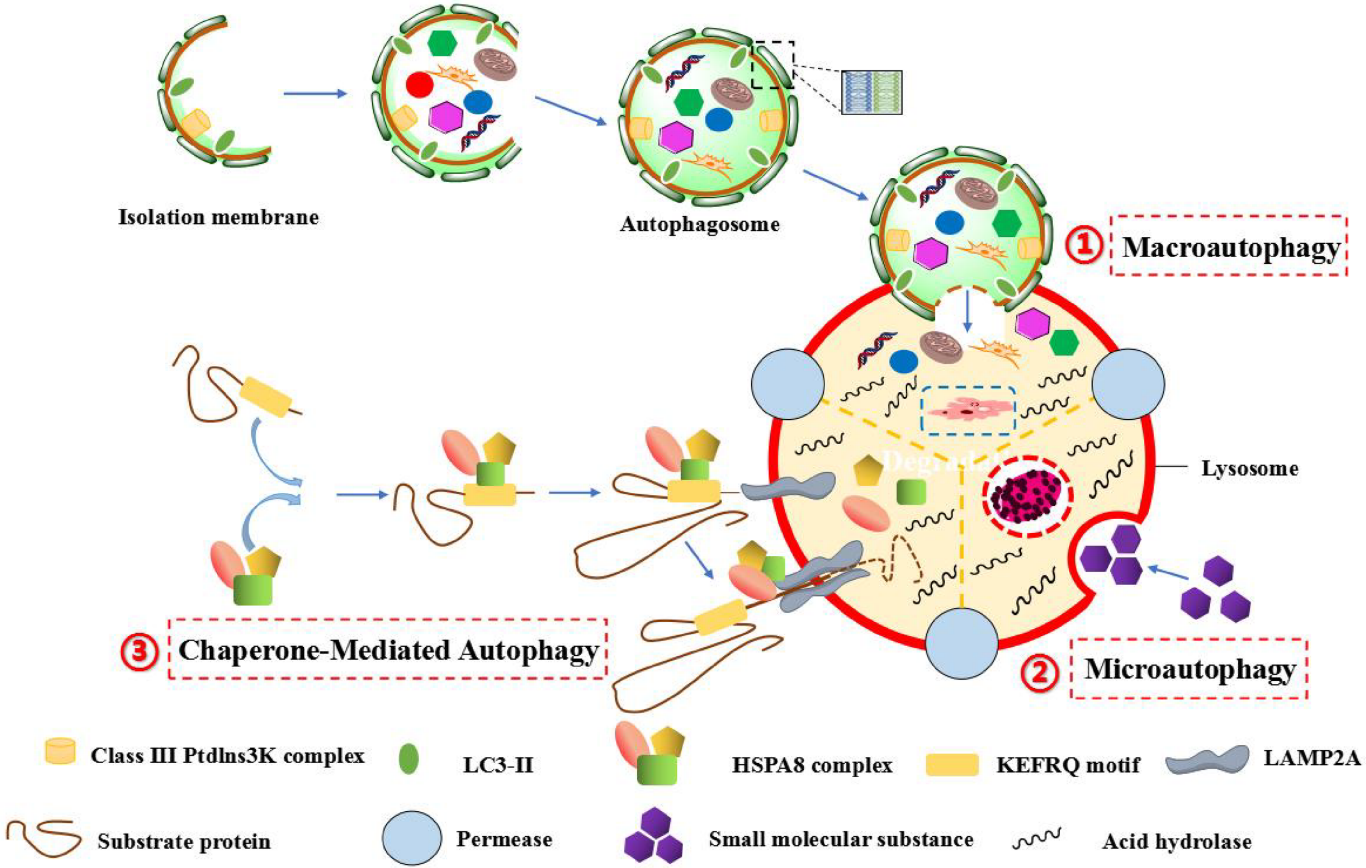
The autophagic process of three different forms in mammalians. (1) Macroautophagy, which is characterized by the formation of autophagosomes with a double-layer membrane structure engulfs intracellular macromolecular substances in a wrapped manner, and the autophagosome eventually fuses with lysosome to form autophagic lysosome, and the inclusion bodies are degraded by hydrolytic enzymes in lysosome. (2) Microautophagy, which is characterized by the specific organelles are directly engulfed in an invaginated manner by the deformation of lysosomal or vacuole surface, and the lysosomal membrane inwardly folds to crush the contents. (3) Chaperone-mediated autophagy (CMA), which is characterized by the specific amino acid sequence (such as KEFRQ motif) of soluble protein is identified and combined by a chaperone-dependent protein (such as HSPA8 complex), and transfer it to lysosomes *via* the receptor LAMP2A on the lysosomal membrane, so as to allow misfolded proteins to undergo defolding and complete degradation by hydrolytic enzymes in lysosome.

In 1962, Ashford and Porter found that a appearance of “eating yourself” into cells, so as to produce the word of “autophagy”. It means that the autophagosome is formed by the double membrane, which is dropped from the ribosome free attachment area of rough endoplasmic reticulum (ER), wrapping organelles, proteins and other components to be degraded in cell. Subsequently, autophagosome merges with lysosome to constitute autolysosome, which resolves their contents to realize the metabolic demands of cells and the update of some organelles (1). In eukaryotic cells, autophagy can be divided into three major categories: megaautophagy, microautophagy and chaperone-mediated autophagy (CMA). Megaautophagy is the main autophagic pathway, damaged organelles or unused proteins are removed through phagocytosis. The process is that the autophagic cell firstly engulfs the macromolecular material, the autophagosome is formed around the macromolecule, and the autophagosome passes through the cytoplasm and merges with lysosome, then the hydrolase in lysosome degrades the inclusion. However, microautophagy is different from megaautophagy. It first directly wraps micromolecules into lysosomes by the form of invagination, and uses lysosomal membranes to fold inward to crush micromolecules. CMA is a very complex and specific pathway, which mainly recognizes and binds soluble proteins with specific sequences of amino acid (such as KEFRQ motif) through chaperoned-dependent proteins (such as HSPA8 complex), and transfers them to lysosome *via* receptor LAMP2a on lysosomal membrane, so as to be degraded by hydrolase (2–5). Moreover, macroautophagy can also be subdivided into ontological autophagy and selective autophagy according to different targets. Ontological autophagy generally degrades the entire target, such as viruses and infected cells. Selective autophagy refers to selectively degrade specific targets, which can be subdivided into different types, including mitochondrial autophagy, lipid autophagy, peroxisome autophagy, chloroplast autophagy, ribosome autophagy, and so on (6) (Figure 1).

In the 1990s, Yoshinori Ohsumi (a Japanese scientist) and his team found multiple autophagy-related genes (ATGs) in the model of yeast, and they found that Almost all functional congeners of yeast ATGs were found in higher eukaryotes. In 2003, Klionsky et al. named these genes the Atg genes and studied the interactions between the encoded proteins and their autophagic functions. Subsequently, in April 2005, a new journal, Autophagy, was published that was edited by Klionsky, so that the number of autophagy-related research papers, which indexed by PubMed, has increased each year. Notably, on October 3, 2016, Yoshinori Ohsumi (a Japanese scientist) was bestowed the nobel prize in physiology or medicine, for his work on “discovering the mechanism of autophagy” (7). On the basis of Yoshinori Ohsumi's research, autophagy, as a novel type of programmed death mode (type-II programmed death), is different from type-I programmed death, has attracted more and more attention from researchers in various fields. Research on autophagy-related diseases and autophagic mechanisms of various types have also been carried out successively. With the deepening of research, it has been revealed that autophagy impacts the occurrence and development of tumors, neurological diseases, aging, immune and other diseases (8). Notably, the relationship between autophagy and cardiovascular disease has been paid more and more attention. Cardiovascular disease is one of the major cause of death on a global scale. Myocardial cells, as the basic cell of cardiovascular system, are in the post mitotic state, which means that they can highly rely on complete autophagy and mitosis to maintain their physiological functions and resist harmful damage. When cells are exposed to the environment of inflammation, hypoxia, lipoprotein oxidation, ER stress and the generation of reactive oxygen species (ROS), the process of autophagy can be triggered. By degrading damaged organelles and inhibiting cell apoptosis, autophagy can provide energy and materials for protein synthesis and biofilm in the “hunger” state, so as to improve cellular survival, while the process is particularly important in the occurrence and development of cardiovascular diseases.

## Relationship between cardiovascular diseases and autophagy

Many literature have relvealed that autophagy is closely associated with the occurrence and development of atherosclerosis, myocardial ischemia/reperfusion (I/R), heart failure and other diseases.

### Atherosclerosis and autophagy

The atherosclerosis is started with the damage of vascular endothelial cells, and smooth muscle cells play an important role in the stability of plaque, while macrophages exist an important function in the stability of mature plaque and the formation of thrombosis. Studies have found that autophagy all occurs in these atherosclerosis-related factors. Among them, moderate autophagy can protect cells from apoptosis and necrosis by degrading damaged structures in cells, stabilize plaque and inflammatory reaction, so as to delay or reduce the occurrence and development of atherosclerosis. However, the abnormality of autophagy is not conducive to the stability of plaque, and promotes inflammatory reaction, which cause the occurrence and development of atherosclerosis (9, 10). The pathological attack of coronary artery occlusion can cause the ischemic stress to myocardial cells, especially the lack of nutrients and the production of ROS.

### Myocardial I/R and autophagy

Following with the adaptive period of reperfusion, the situation of myocardial I/R can be occurred. Plenty of evidences indicate that autophagy is closely associated with the etiology of myocardial ischemia-reperfusion injury (IRI). However, autophagy plays a role of “double-edged sword” in the process of myocardial I/R, which has been widely confirmed. On the one hand, autophagic flow increases significantly during myocardial ischemia-injury, and autophagy plays a protective role at this time. On the other hand, during the adaptive period of myocardial-reperfusion, autophagy is overly activated to produce damage, so as to cause the death of myocardial cells (11, 12).

### Heart failure and autophagy

Heart failure is a progressive disease. Its pathological changes are mainly characterized by hypertrophy of myocardium and reduction of myocardial contractility. Although autophagy can effectively manage the myocardium at the physiological level, the effect of autophagy on the pathological condition of heart failure is controversial. Oka et al. (13) found that in myocardial cells lacking lysosomal deoxynucleotidase, giving overloaded stimulation for 10 days could induce myocarditis and dilated cardiomyopathy, and eventually caused heart failure, indicating that inhibition of ATG leaded to myocardial remodeling. Ucar et al. (14) attached importance to the role of microRNAs in the heart failure-induced autophagy. They found that the expression of mi RNA-212/132 in hypertrophic cardiomyocytes was up-regulated, the degree of myocardial hypertrophy in the gene knockout mice was obviously lower than that in the control group during heart failure, and delayed the development of heart failure, indicating that mi RNA-212/132 inhibited autophagy and promoted the process of myocardial hypertrophy. In the experiment of injecting diphtheria toxin into mice, De Meyer et al. (15) found that myocardial cells appeared characteristic features of autophagy-induced cell death after 7 days, including increase of lysosome and accumulation of autophagy. After 14 days, heart failure occurred in mice. These evidences suggest that autophagy plays a dual role in heart failure. Autophagy plays an adaptive role in progressive heart failure and protects myocardial cells. In the late period of heart failure, substances and injury myocardial cells can be overly removed *via* autophagic pathway.

### Dual role of autophagy in the occurrence of cardiovascular diseases

As mentioned above, autophagy is not absolutely beneficial in the occurrence of cardiovascular diseases. It is like a “double-edged sword” with dual role. On the one hand, physiological autophagy has a protective effect on the maintenance of cardiovascular function. Various stressful reactions, including myocardial IRI, chronic hypoxia and so on, can induce autophagy in myocardial cells, reasonably regulating autophagy can help myocardial cells adapt to the environmental changes, so as to resist damage caused by various adverse stimulation. On the other hand, over-expression of autophagy can also induce cells to enter the procedure of death, so as to promote the development of disease. Therefore, cardiovascular-related cells are benefited from the activation of autophagy, and inhibition of autophagy may promote the progress of disease. Meanwhile, excessive autophagy can induce autophagic cell death. In view of this controversy, it is particularly important to regulate autophagy at a reasonable activity of autophagy. Notably, autophagy modulates the process of cardiovascular diseases through many ATGs and signaling pathways.

Therefore, in order to further epitomize the recent advances in the autophagic research in cardiovascular disease, we will elucidate the regulatory mechanism of autophagy, focusing on the role of autophagy in the occurrence and development of cardiovascular diseases and its signaling pathways. Subsequently, we will discuss the potential treatments of cardiovascular diseases *via* targeting those relevant pathways. We hope to provide a comprehensive perspective and guidance for further understanding the potential mechanisms of autophagy in cardiovascular diseases, and a unique perspective on autophagic regulation as a therapeutic regimen strategy for cardiovascular diseases.

## Role of main mechanism autophagy genes in cardiovascular disease

### Core molecular mechanism of autophagy

Generally, the fundamental process of autophagy includes the following steps: the formation of autophagic precursors (the membrane structure of ER and mitochondria in the cytoplasm will form autophagic precursors with double membrane cup-shaped separators), formation of autophagic vesicles (the septum extends continuously and gradually encloses the garbage in the cytoplasm to form a closed autophagic vesicle), formation of autolysosome (autophagosome merges with lysosome to form autolysosome), degradation (the encapsulated substances are degraded through the acid hydrolase in lysosome, and the degraded products are recycled) (16). Autophagy is very conservative in evolution, and its occurrence and development are regulated by different ATGs. ATG protein plays an important role in the formation of autophag bodies and the transmission of autophagy-loaded lysosomes. It can be divided into five complexes: (1) RB1-inducible coiled-coil protein 1 (FIP200), ATG101, ATG13 and unc-51-like kinase 1 (ULK1) complex. (2) Class III PI3K (PI3KC3) complexes, the catalytic subunits of vacuolar proteins sorting 34 (VPS34), Beclin 1 and p115, connected by ATG14 or ultraviolet-resistant binding gene (UVRAG) proteins. (3) Two ubiquitin-type proteins and conjugation systems: ATG12-ATG5-ATG16L complexes and UB-type proteins of the ATG8 family (ATG8s), which form conjugates in the presence of phosphate ethanolamine (PE) membranes. (4) ATG18/WIPI (WD repeat phosphoinositol interaction) and ATG2 proteins. (5) ATG9, the only multi-hop transmembrane protein involved in vesicle transport (17, 18). At present, at least 30 kinds of ATG proteins have been identified in yeast, human and mice (Table 1).

**Table 1:** Comparison of autophagy-related genes in yeast, human and mice.

Yeast	Human	Mice
ATG 1	ULK 1	
ATG 3	HsATG 3/HsAPG 3	MATG 3/MAPG 3
ATG 4	HAtg 4A/HsATG 4A	
	HsAPG 4A/autophagin-2	
	HAtg 4B/HsATG 4B	
	HAPG 4B/autophagin-1	
	HAtg 4C/HsAUTL 1	
	autophagin-3	
	HAtg 4D/ autophagin-4	
ATG 5	HAtg 5/HAPG 5	
ATG 6	Beclin 1	
ATG 7	HAtg 7/HsGSA 7/HAPG 7	MATG 7/MAPG 7
ATG 8	LC 3	
	GABARAP modifier	
	GATE-16	
	ATG 8L/APG 8L	
ATG 10	MATG 10/MAPG 10	
ATG 12	HAtg 12/HAPG 12	MATG 12/MAPG 12
ATG 16		ATG 16L/APG 16L

**Figure 2:**
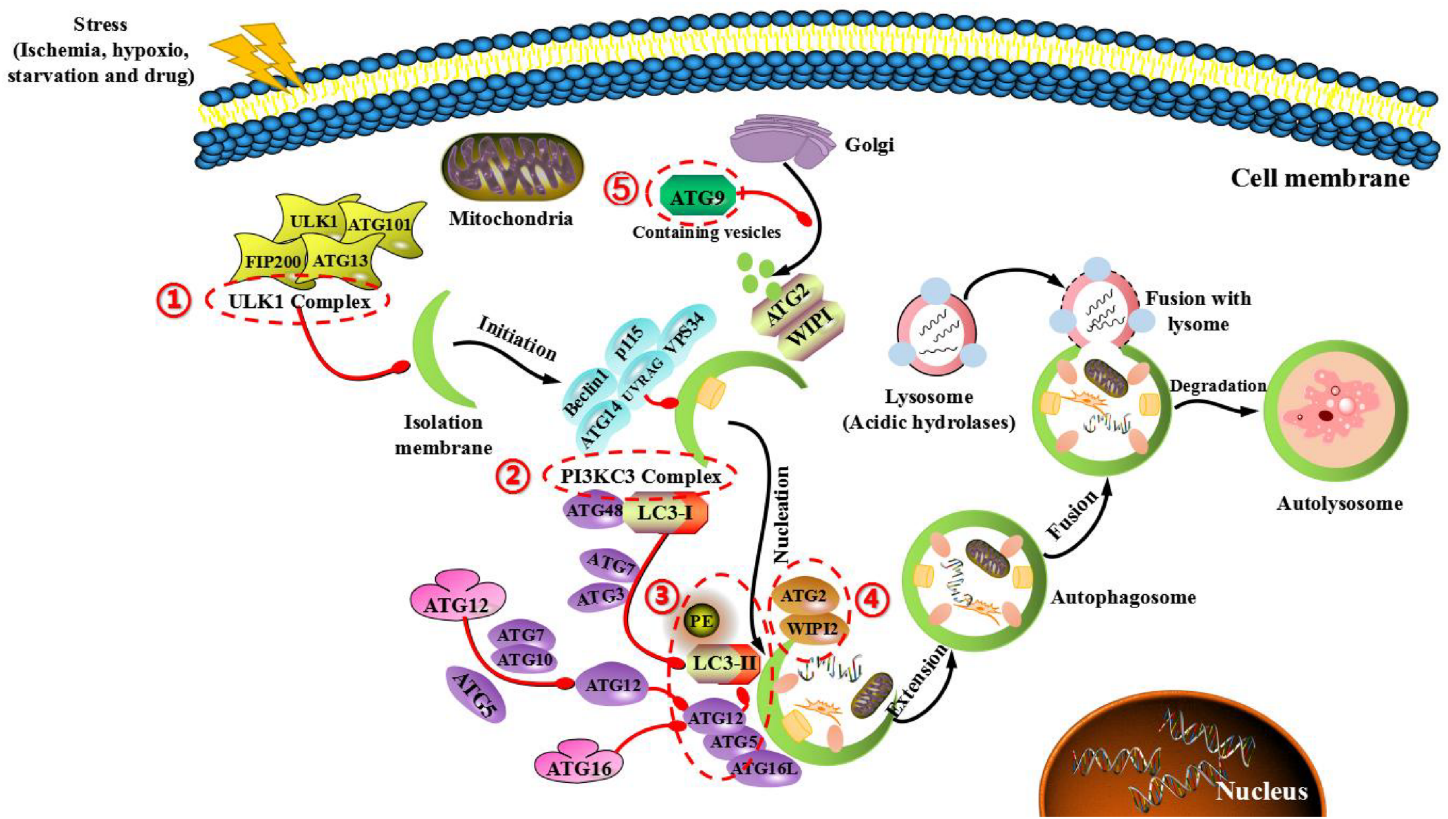
Main mechanism of autophagy in mammals and target genes for treating cardiovascular diseases. The main mechanism is that ATG protein forms five functional groups (indicated in the red box). (1) ULK1 complex, composing of FIP200, ULK1, ATG13 and ATG101, which the mTOR complex of negative regulation. (2) BECLIN1-III PI3K complex, composing of BECLIN-1, VPS34, P115, AMBRA1 and ATG14 control the nucleation phase of autophagy. (3) Two full sample combination systems (ATG12-ATG5 and LC3 systems), which regulates the structure of autophagic precursor and autophagic body. (4) WIPI1/2 and ATG2 complexes control the extending autophagosomal membrane. (5) ATG9 retrieval complex, which the only multi-hop transmembrane protein involved in vesicle transport.

In addition, autophagy can be divided into five stages, and these ATG proteins also play an important role in each stage (Figure 2). (I) Initial stage of autophagy: under the action of autophagy-induced signals (starvation, hypoxia, pharmaceutical intervention and so on), a large number of free membranes from ER, mitochondria and other organelles in the cytoplasm can form an autophagic precursora (like bowl shaped structure), and ATG1 complexes (including ATG1/ULK1, ATG13 and ATG17/FIP200) are used to mediate the initiation of autophagy. At this time, the activation of mammalian target of rapamycin complex 1 (mTORC1) signal is inhibited, the phosphorylated level of ATG13 is reduced, and the ATG13-ATG1-ATG17 complex forms and starts the process of autophagy. (II) Nucleated stage of autophagic vesicles: Vps34 (PI3K)-ATG6 (Beclin-1) complex can not only act on the nucleation of membranous vesicles, so as to mediate the formation of autophagic precursor structure by regulating protein kinase Vps15, but also gather ATG12-ATG5 complex, ATG16 polymer and LC3 molecule to promote the expansion of phagocytes. (III) Extended stage of autophagosome: in the process of autophagosomal membrane extending to both ends, autophagy mainly depends on two ubiquitinated connective systems. One is ATG12-ATG5-ATG16 ubiquitinated connective system. Among them, ATG12 is first activated by ATG7, then transported by ATG10 and bound to ATG5. Subsequently, ATG12-ATG5 complex is combined with ATG16 to generate a ternary complex of ATG12-ATG5-ATG16, which is located on the outer membranous surface of autophagic precursor structure. The other is the ubiquitinated connective system of microtubule associated protein I light chain (LC3). After the formation of LC3 precursor, ATG4 modifies it into cytosolic soluble LC3-I, and under the action of ATG7 and ATG3, PE is covalently added to the glycine residue at the carboxylic terminus of LC3-I to form liposoluble LC3-PE (LC3-II), and LC3-II can be stably bound to the double membrane of autophagic vesicles. Notably, LC3-I is usually dissociated in the cytoplasm, and once transformed into LC3-II, it can specifically bind to the double membrane of autophagosome. Therefore, LC3-II is often used as a marker of autophagic formation, and it is also an important regulatory protein of multiple signaling transduction located on the membrane of autophagic vesicle. These two linkage systems interact and regulate reciprocally, and both need the participation of ubiquitinated activating enzymes E1 and E2, so as to jointly promote the extension of autophagosomal membrane. (IV) Maturated stage of autophage: during the extended process of autophagosomal membrane, components to be degraded in the cytoplasm are continuously pulled into the membrane, and a sealed autophagosome with spherical structure is formed. Subsequently, under the action of endosomal sorting complex required for transport (ESCRT) and monomer GTP enzyme (RabS), autophagosomes fuse with lysosomes to form autolysosomes through microtubule skeleton. Lysosome-related proteins (include LAMP1, LAMP2 and UVRAG) also involve in this process. (V) Degradated stage of autolysosome: the membrane of autolysosome begins to crack, then the encapsulated contents are degraded under the action of acid hydrolase in lysosome. Amino acids, fatty acids and other products are generated from the process of degradation and transported to the cytoplasm, so as to provide nutrition and energy for cellular growth, while residues are expelled or retained in cytoplasm to maintain the stability of internal environment and the renewal of organelles. During this period, ATG22 is directly involved in the transport of some amino acids. ATG12 and ATG15 may be involved in the cleavage of autolysosomal membrane, while ATG1 and ATG13 are involved in the transport of acid hydrolase in lysosome (19).

With the help of ATGs protein complex, the entire process of cell autophagy was completed, which jointly promotes the transport of cargoes in autophagosome to autolysosome and the degradation *via* the action of acid hydrolase, then the degraded products are transported to the cytoplasm for cellular recycling. Moreover, it is extremely necessary to have a deeper understanding of the potential mechanism of autophagy before deciding whether to induce or inhibit autophagy to treat human diseases in the future.

### Role of autophagy mechanism genes in the diagnosis of cardiovascular disease

At present, the recurrence rate of patients with cardiovascular diseases is high and the survival rate is low. It is very important to find practical biomarkers for predicting the therapeutic risk and prognosis of patients with cardiovascular diseases (20). Some critical ATGs can be used as prognostic biomarkers of cardiovascular diseases. The use of autophagy-related markers will benefit the prognosis of cardiovascular diseases and broaden the therapeutic methods.

**Figure 3:**
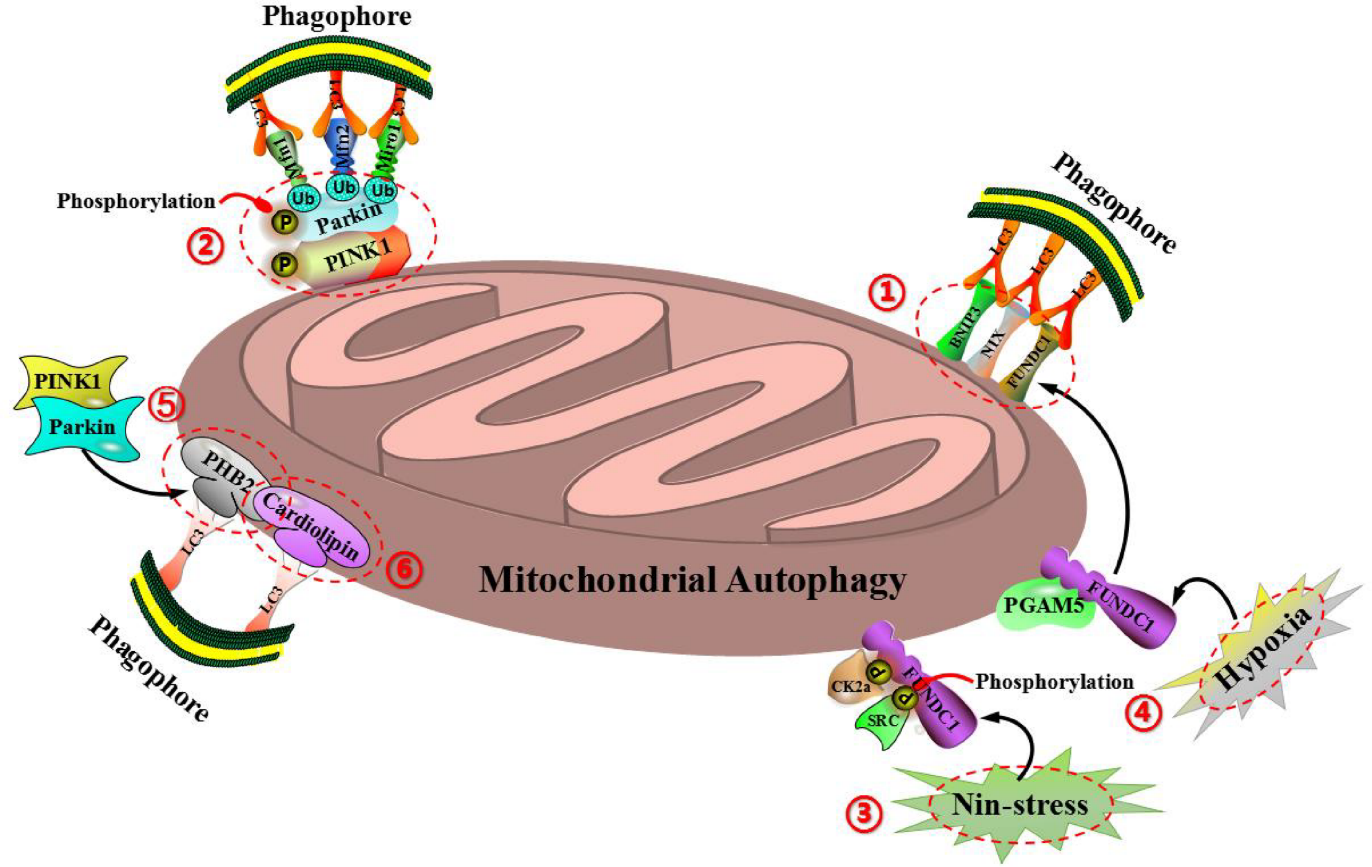
Molecular mechanism of mitochondrial autophagy regulation can be divided into two types: ubiquitin-dependent mitochondrial autophagy (including PINK1 and Parkin) and ubiquitin-independent mitochondrial autophagy (including BNIP3, NIX, FUNDC1, PHB2, cardiolipin and so on). (1) BNIP3, FUNDC1, and NIX are all located on the outer membrane of mitochondria and can bind directly to LC3-mediated mitochondrial autophagy *via* the domain of LC3 interaction (LIR). (2) BNIP3 can also inhibit the proteolysis of PINK1, so as to cause the accumulation of PINK1 on the outer mitochondrial membrane, thereby promoting PINK1/Parkin-mediated mitochondrial autophagy. (3) Under the condition of non-stress, FUNDC1 is phosphorylated by protein kinase CK2α in the region of Ser-13 and phosphorylated by protein kinase SRC in the region of Tyr-18, so as to inhibit the interaction of LC3 with FUNDC1, thereby preventing the occurrence of mitochondrial autophagy. (4) Under the condition of mitochondrial membrane potential loss or hypoxia, protein phosphatase PGAM5 can interact with FUNDC1 to prevent CK2α and SRC kinase to combin with FUNDC1, resulting in the dephosphorylation of FUNDC1, so as to enhance the interaction between FUNDC1 and LC3, and induce mitochondrial autophagy. (5) As an important receptor for mitochondrial autophagy, the inner mitochondrial membrane protein PHB2 can mediate mitochondrial autophagy *via* the domain of LIR, and bind to the autophagic membrane-related protein LC3 during the process of mitochondrial depolarization and proteasome-dependent outer membrane rupture, which is closely associated with the PINK1/Parkin signaling pathway. (6) Cardiolipin (CL), as a phospholipid in the inner mitochondrial membrane, can be externalized to the outer mitochondrial membrane when mitochondria are damaged, while the redistribution of CL and its interaction with LC3 initiate a signaling cascade and mediate mitochondrial autophagy.

Mitochondrial autophagy is one of the most familiar autophagies in cells. Plenty of studies have shown that under normal situation, mitochondrial autophagy can maintain the stability of mitochondrial structure and function in cells, so as to ensure the normal operation of cardiac function. Abnormal mitochondrial autophagy is closely related to cardiovascular diseases, including hypertension, ischemic heart disease, atherosclerosis, diabetes cardiomyopathy, heart failure and so on (21). Therefore, mitochondrial autophagy plays an important role in the prevention and treatment of cardiovascular diseases. At present, mitochondrial autophagy can be mainly divided into two types according to different regulatory pathways: The one is ubiquitin-dependent mitochondrial autophagy, which is mainly mediated by PTEN induced putative kinase 1 (PINK1) and E3 ubiquitin ligase (Parkin) (22). The other is ubiquitin-independent mitochondrial autophagy, which is mainly mediated by BCL2 interacting protein 3 (BNIP3), NIP3-like protein1 X (NIX), FUN14 domain containing protein 1 (FUNDC1), anti-proliferative protein 2 (PHB2), cardiolipin (CL) and so on (23). Notably, BNIP3, FUNDC1 and NIX are located in the outer membrane of mitochondria. BNIP3, a homologous protein of Bcl-2, is located in mitochondrial outer membrane and induces mitochondrial autophagy and apoptosis (24). Under the inducation of hypoxia, BNIP3, as a receptor of mitochondrial autophagy, can directly bind to LC3-mediated mitochondrial autophagy through the LC3 interaction (LIR) domain (25, 26). BNIP3 can also inhibit the proteolysis of PINK1, which leads to the accumulation of PINK1 on the mitochondrial outer membrane, so as to promote PINK1/Parkin-mediated mitochondrial autophagy (27). FUNDC1 is a novel mitochondrial outer membrane protein, which starts mitochondrial autophagy by binding LIR domain with LC3, and belongs to the pathway of receptor-mediated mitochondrial autophagy (28, 29). FUNDC1-mediated mitochondrial autophagy can be regulated by reversible phosphorylation. Under non-stressful conditions, FUNDC1 is phosphorylated by protein kinase CK2α at the regionof Ser-13 and phosphorylated by protein kinase SRC in the regionof Tyr-18, then inhibits the interaction between LC3 and FUNDC1, so as to prevente the occurrence of mitochondrial autophagy (28). Under the condition of mitochondrial membrane potential loss or hypoxia, protein phosphatase PGAM5 can interact with FUNDC1 to prevent CK2α and SRC kinase to combin with FUNDC1, leading to the dephosphorylation of FUNDC1, so as to enhance the interaction between FUNDC1 and LC3 and induce mitochondrial autophagy (30). Mitochondrial outer membrane protein NIX, also be called as BNIP3L, is a member of the Bcl-2 family (31). As a receptor of mitochondrial autophagy, NIX can directly bind to LC3 through the LIR domain to mediate mitochondrial autophagy. NIX exists a key role in programmed mitochondrial autophagy during the process of cell differentiation. The lack of NIX promotes the accumulation of injured mitochondria in cells, which leads to increase the apoptosis and developmental defects, but the signaling cascade of NIX-mediated mitochondrial autophagy is still unclear (32, 33). Interestingly, PHB2 and CL are located in the mitochondrial inner membrane. As an important receptor of mitochondrial autophagy, mitochondrial inner membrane protein PHB2 mediates mitochondrial autophagy through LIR domain and binds to autophagic membrane-related protein LC3 during mitochondrial depolarization and proteasome-dependent outer membrane rupture (34). Notably, the proteasome-dependent mitochondrial outer membrane rupture is closely associated with PINK1/Parkin signaling pathway (35, 36). Cardiolipin (CL), as a phospholipid in mitochondrial inner membrane, can be externalized to mitochondrial outer membrane when mitochondria is damaged. The redistribution of CL and its interaction with LC3 initiate signaling cascade and mediate mitochondrial autophagy (37) (Figure 3).

Increasing reports have shown that CMA can not only participate in the molecular regulation of various diseases including cardiovascular diseases, but also maintain intracellular energy homeostasis *via* the timely degradation of enzymes involved in glucose and lipid metabolism (38). Therefore, CMA may become an attractive therapeutic target for cardiovascular diseases. It is well-known that hypoxia is not only an important factor in myocardial infarction and I/R injury, but also a major precedent cause of heart failure. Due to CMA is a turnover process of partner-dependent selective cytosolic protein, its targeting to specific proteins can be recruited into lysosomes for degradation (39). It has been shown that enhancing protein degradation mechanisms is beneficial in a variety of heart disease models, including myocardial infarction and I/R injury (40). And Ghosh et al. (41) hanve further found that CMA can enhance the degradation of targeted specific proteins through the over-expression of lysosome-associated membrane protein 2a (Lamp2a), so as to have a protective effect on hypoxia-induced cardiomyocyte damage. Another study have demonstrated that CMA can be used for the degradation of lysosomes in cardiomyocytes by targeting PARP1, and finally inhibit the apoptosis of cardiomyocytes by promoting the degradation of PARP1 protein (42). Thus, these evidences reveal the interesting possibility that the activation of CMA may provide cardioprotective therapy for ischemic heart disease. Clinically, risk factors affecting the development and progression of atherosclerosis can include systemic parameters, such as hyperlipidemia, hypertension, insulin resistance, and age. At the level of local vessel, atherosclerosis involves a complex interplay between macrophage infiltration, pro-inflammatory activation, smooth muscle cell dedifferentiation into secretory and inflammatory phenotypes, and induction of cell death, while the co-occurrence of these events promotes the formation of large necrotic cores and enhances the degradation of matrix degradation, so as to weaken atherogenic plaques and increase their risk of rupture and subsequent thromboocclusion (43). Due to CMA is a lysosome-degradation-selective type of intracellular protein that can decrease with age and is inhibited by excess lipid (44). As Qiao et al. (45) have found that the lack of CMA may promote lipid accumulation in macrophages by regulating enzymes involved in lipid metabolism, which in turn are typical components in atherosclerotic plaques, suggests that CMA may represent a novel therapeutic target to alleviate the progression of atherosclerosis by promoting lipid metabolism. And Matute et al. (46) have demonstrated the protective effect of CMA on atherosclerosis by related experiments, and have demonstrated the value of CMA reactivation in retarding disease progression and reducing clinical events. It can be seen that although CMA can be declined with age and continuous high-fat diet, the stimulation of CMA can play its therapeutic value by reducing the risk of vascular events and improving the severity and progress of disease. Another study have shown that when the function of CMA is damaged during the progression of atherosclerosis, it can increase the activation of inflammatory body NLRP3 and the secretion of IL-1β, so as to promote the progress of vascular inflammation and atherosclerosis (47). And the research of Qiao et al. (48) have also revealed that the new mechanism of inflammatory body NLRP3 is regulated in macrophages and atherosclerosis, so as to provide new insights into the role of autophagy-lysosome pathway in atherosclerosis, indicating that the pharmacological activation of CMA may provide new treatment strategies for the atherosclerosis and other inflammatory body NLRP3/IL-1β driven diseases.

Melatonin (Mel) is an endogenous hormone secreted mainly by the pineal gland that acts as a buffer in the immune system and shows the characteristics of pro-inflammatory and anti-inflammatory. It can not only enhance the body's defense mechanisms against the invasion of pathogenic microorganisms, but also exhibits appropriate anti-inflammatory effects in many low-grade chronic inflammatory diseases including atherosclerosis (49, 50). It has been reported that Mel exerts its vascular protective function through the regulation of autophagy (51, 52). Increasing evidence suggests that innate immune cells can develop a sustained pro-inflammatory phenotype following short exposure to various stimuli during the development of atherosclerosis (53). In this regard, it has been observed that monocytes/macrophages are the most abundant inflammatory cells in atherosclerotic plaques (54). Although statins are commonly used clinically to control the level of cholesterol, macrophages are the important contributors to the residual inflammatory risk associated with recurrent atherothrombotic events (55, 56). In addition, atherosclerotic macrophages are characterized by defects in the autophagic process, despite the presence of many autophagy-stimulating factors (57, 58). Galectin-3 (Gal-3), a member of the galectin family, is currently considered a potential biomarker of cardiovascular inflammation (59). In a prospective cohort study involving 782 angiographic atherosclerosis patients, atherosclerosis patients at low level of Gal-3 had a better prognosis compared with higher level of Gal-3 (60). A research has shown that Gal-3 can promote the uptake of lipoproteins in foam cells, and aggravate oxidized low-density lipoprotein (ox LDL) mediated endothelial damage *via* inducing inflammation (61–63). And recent studies have also shown that Gal-3 directly or indirectly interacts with the inflammasome NLRP3 to amplify the inflammatory response, so it is considered a critical promoter of atherosclerotic inflammation (64–66). Thus, inhibition of Gal-3 may be a potential therapeutic strategy to alleviate atherosclerotic inflammation (64, 67). However, the current inhibitors of Gal-3 can only act extracellularly or on the cellular membrane, and can not directly enter the cell. Notably, Mel is a small endogenous biological molecule that has a short biological half-life, low toxicity, high bioavailability compared with traditional drugs, and it can enter the cells freely. As Wang et al. (68) have conducted *in vivo* and *in vitro* experiments by using respectively ApoE mice fed with high-fat diet (HFD) and THP-1 macrophages, and they have demonstrated that Mel can promote autophagy and inhibit atherosclerotic inflammation by down-regulating Gal-3, which means that Mel is expected to be a therapeutic strategy for atherosclerosis.

Following with the development of modern science and technology, the perfect integration of computer technology and bioinformatics provides a powerful tool for screening autophagy-related genes to affect the occurrence and development of cardiovascular diseases. Comprehensive bioinformatics analysis showed that 60 gene-related autophagy processes have different expression patterns in normal heart tissues and dilated cardiomyopathy (DCM) tissues. Based on Lasso Cox regression algorithm, eight ATGs (BAD, DICER1 HSF1, HSPG2, PLEKHF1, TRIM65, TFEB, VDAC1) were fond to be closely related to the overall survival (OS) rate of DCM. They may be underlying markers for predicting the danger signals of organic changes in heart disease (69).

However, the methods for monitoring autophagy activity are complex and are not always directly related to the increase of autophagic activity. Hence, when selecting diagnostic markers for cardiovascular treatment, it is necessary to combine the changes of autophagic level with the detection of autophagic structure and the monitoring of autophagic flow, so as to provide a more comprehensive and reliable strategy.

## Role of cascade autophagy in cardiovascular disease through regulatory signals

The molecular mechanism of cardiovascular disease includes two important mechanisms: (1) Many molecular factors are associated with infection caused by environmental and metabolic factors. (2) Mutation in cardiovascular diseases-related sites. These two mechanisms are closely associated with several key signal pathways that affect the process of cardiovascular diseases. Hence, this section focuses on signaling cascades, namely PI3K/AKT/mTOR, epidermal growth factor (EGFR) and insulin-like growth factor (IGF), AMPK/mTOR, mitogen-activated protein kinases (MAPKs), p53, Nrf2/p62, Wnt/β-catenin and nuclear factor-κB (NF-κB) pathways. We will also discuss the impact of autophagy on cardiovascular disease through cross-interference with the above pathways and its potential application in the treatment of cardiovascular disease.

### PI3K/Akt/mTOR signaling pathway

PI3K/Akt/mTOR pathway is a highly conserved pathway, which is mainly consisted of three active molecules: mammalian phosphatidylcreatine kinase (PI3K), protein kinase B (PKB/Akt) and rapamycin-like target protein (mTOR). It has been proved to play an important role in modulating cell growth, proliferation, apoptosis, oxidation and angiogenesis. PI3K/Akt/mTOR pathway is activated highly in cardiovascular diseases, and inhibition of mTOR plays a protective role in myocardial cell damage (70).

PI3K is a dimer consisted of regulatory subunit p85 and catalytic subunit P110. When bound to growth factor receptors (such as IGF-1 or EGFR), it can change the structure and activation of Akt protein, and inhibit or activate the activity of several downstream substrates (such as apoptosis-related proteins Caspase-9 and Bad) in the form of phosphorylation, so as to regulate cellular proliferation, differentiation, apoptosis, migration and other phenotypes (71). In addition, mammalian rapamycin target protein (mTOR) is also one of the downstream targets in PI3K/Akt pathway. mTOR is one of the most studied target proteins in autophagic regulation, and exists two complexes in cells: mTORC1 and mTORC2 (72). Under the condition of adequate nutrition, mTORC1 directly interacts with or phosphorylated ULK1, leading to the inhibition of autophagy. On the contrary, under stressful conditions (such as low-nutrition and hypoxia), mTORC1 can be inhibited, dephosphorylation of ULK1 is activated and transferred from cytosol to autophagosome, so as to induce autophagy (73).

A large number of documents have shown that PI3K/Akt/mTOR pathway affects the process of atherosclerosis, heart failure, myocardial I/R and other diseases. The pathogenesis of atherosclerosis is mostly related to vascular endothelial injury, inflammatory reaction and fat metabolism disorder (74). It is found that oxidized low-density lipoprotein (ox LDL) can inhibit the phosphorylation of Akt and eNOS in PI3K/Akt/eNOS signaling pathway, accelerate endothelial cell apoptosis and inflammatory reaction, so as to accelerate the formation of atherosclerosis (75). Xing et al. (76) have found that salidroside can activate eNOS, enhance the release of vascular endothelial relaxing factor NO, improve endothelial function, reduce the inflammation of aortic sinus injury, and reduce the area of atherosclerosis. Using LY294002 can inhibit the salidroside-induced phosphorylation and expression of eNOS. Besides, Dou et al. (77) have found that regulation of the PI3K/Akt/eNOS signaling pathway can improve the lipid metabolism, glucose tolerance and insulin sensitivity of apolipoprotein edeficient mice, and has the effects of protecting vascular endothelium and anti-atherosclerosis.

Heart failure is the terminal period of many cardiovascular diseases. PI3K/Akt signaling pathway can inhibit myocardial cell apoptosis through downstream target-mediated multiple apoptosis factors. Bad is the downstream target protein of Akt, which often exists in the form of dimer (Bcl-2/Bad, Bcl-xl/Bad). Activated Akt can phosphorylate Ser136 at the Bad site, resulting in 14-3-3 protein binding to Bad, so as to inhibit the proapoptotic effect of Bad and dissociate Bcl-2 to play a role of anti-apoptosis. Meanwhile, Akt can also prevent the release of mitochondrial cytochrome C, so as to affect the activity of Caspase-3, which the main executor of apoptosis (78, 79). Hong et al. (80) have found that tanshinone IIA can enhance the phosphorylation of Akt, inhibit the expression of Bax, reduce the release of cytochrome C, and increase the expression of Bcl-2, so as to protect myocardial cells. This find suggests that effective activation of Akt signaling pathway benefits the survival of myocardial cells, reduce their apoptosis, and then alleviate heart failure. Lin et al. (81) have found that activation of PI3K can promote the high expression of growth factor receptor binding protein 14 (Grb14) in the heart, and can significantly improve the heart function of mice with heart failure. However, protective effect of Akt on myocardial cells is transient. If the injurious factor continues to exist and Akt is activated for a long time, it will cause the pathological hypertrophy of myocardium, myocardial fibrosis and so on, resulting in the deterioration of cardiac function. Wohlschlaeger et al. (82) have found that phosphorylation of myocardial Akt in patients with advanced heart failure is increased, and phosphorylation of Akt is decreased significantly after treatment with left ventricular assist device and improvement of cardiac function.

Vascular smooth muscle cells are closely associated with the instability and rupture of atherosclerotic plaque. Studies have shown that vascular smooth muscle cells can activate PI3K/Akt pathway by secreting basic fibroblast growth factor (bFGF), and reduce myocardial I/R-induced apoptosis and autophagy (83). Cheng Songyi (84) has found that astragaloside A can activate PI3K/Akt pathway, increase the expression level of vascular endothelial growth factor (VEGF), promote angiogenesis after myocardial infarction and reduce heart damage by interfering with myocardial infarction rats and human umbilical vein endothelial cell (HUVEC) with different doses of astragaloside A. Leng et al. (85) used ginsenoside Rg1 to intervene in the model of isoproterenol-induced acute myocardial ischemia. They found that ginsenoside Rg1 could significantly increase the mean blood flow on the myocardial surface, increase the content of serum NO, reduce the levels of creatine kinase (CK) and lactate dehydrogenase (LDH), and increase the expression of eNOS mRNA in myocardial tissue by regulating PI3K/Akt signaling pathway.

### IGF-1 and EGFR signaling pathway

The IGF pathway plays a key role in antiapoptosis, stimulating proliferation, activating angiogenesis, and initiating and maintaining tumorigenesis (86). At present, almost all the researches on this pathway for blood vessels have been completed on animal models. The study has showed that the probability of cardiovascular disease in rodent model is significantly reduced when the level of serum IGF-1 is low. However, there are few clinical studies on serum IGF-1, and the reported results are different. In humans, the deficiency of insulin receptor can cause insulin resistance and diabetes, and the deficiency of IGF-1 increases the risk of atherosclerosis (87). However, it has been reported that IGF-1 can promote the migration and proliferation of human coronary artery vascular smooth muscle cells, and has found that the concentration of IGF-1 in human coronary atherosclerotic plaque vascular smooth muscle cells is significantly higher than that in normal coronary artery vascular smooth muscle cells, and the expression level of IGF-1R mRNA in atherosclerotic plaque vascular smooth muscle cells is also increased (88). Moreove, the IGF2 gene is then transcribed in developmental and tissue-specific sequences, and four promoters (P1–P4) are participated in the alteration of IGF2 expression levels (89). The interruption of IGF2 promoter regulation is a common feature of human cardiovascular diseases, which indicates that frequent deletion of biallelic IGF2 expression due to the deletion of P1 activation can be used as a diagnostic or observational marker for human cardiovascular diseases (90, 91). Different from the high expression of IGF-2 in cardiovascular diseases, and IGF2R also has low expression in cardiovascular diseases. This excess ligand can enhance the binding ability of receptor, and increase the activity of MAPK and PI3K/AKT/mTOR signaling pathways, which are the main regulatory factors of autophagic regulation (92).

EGF receptor (EGFR or HER1) is one of the most related growth factor receptors in hepatocellular carcinoma (HCC). Activating PI3K/Akt/mTOR and RAF/MEK/ERK signaling pathways play an important role in the proliferation and angiogenesis of tumors (93). High levels of EGFR are detected in adult hepatocytes, which indicates that EGFR plays an important role in liver protection and regeneration (94). In addition, EGFR pathway is also participated in proliferation, survival and tumor formation of human HCC cell line *in vitro* (95). Compared with tumors, the study of this pathway in cardiovascular field is less. Research shows that EGFR may be related to heart injury caused by diabetes. Streptozotocin (STZ)-induced type 1 diabetes mice are respectively dealed with EGFR inhibitors AG1478 and 451 for 8 weeks. It is found that diabetes induces the phosphorylation of EGFR and Akt, increases the level of cardiac ROS, and eventually leads to cardiac morphological changes, consisting of cardiac hypertrophy, apoptosis and fibrosis. All these molecular and pathological changes are weakened by EGFR inhibitors. Following with the validation of cultured cells *in vitro*, the pharmacological inhibition of EGFR/Akt or silencing of sh-RNA-EGFR significantly inhibits ROS production induced by high concentration glucose (HG), and the phenomenon of apoptosis respectively occurs in H9C2 cells and primary rat cardiomyocytes. Meanwhile, ROS inhibitor NAC can also reduce the HG-induced injury of myocardial cells. It can be seen that EGFR exisits a critical role in the pathogenesis of STZ-induced myocardial injury and ROS changes in diabetes, indicating that EGFR may be a underlying target for the therapy of diabetic cardiomyopathy (96).

### AMPK/mTOR signaling pathway

AMPK/mTOR pathway activated by adenosine monophosphate (AMP) plays an important role in cell growth, cell proliferation and metabolism, and autophagic regulation (97). More and more literature have shown that AMPK/ mTOR signaling pathway is participated in the process of cardiovascular diseases (98, 99). The formation of VPS34-VPS15-Beclin1 core complex can occur at the initial stage of autophagy. Following with the addition of other autophagy related-protein (Atg14L) to the core complex, type III phosphatidylinositol 3-kinase (VPS34) can gradually activate and produce phosphatidylinositol 3-phosphate (PtdIns3P), which has a significant role in promoting the extension of autophagic vesicles, with phosphatidylinositol (PtdIns) as the substrate. However, when autophagy does not occur, Beclin1 can bind to Bcl-2, thus being in a resting state. Many nutrition and growth related-kinases (such as mTOR, EGFR and so on) can phosphorylate Beclin1, which is the core scaffold protein in VPS34-VPS15-Beclin1 core complex, and control the lipase activity of VPS34 by changing the composition of the complex, so as to regulate the occurrence of autophagy. AMPK is an important kinase in cell energy sensing and cell signal regulation during the process of autophagy. Under stressful condition, the decrease of ATP increases AMP/ATP ratio, which further activated energy-induced kinase, liver kinase B1 (LKB1) and AMPK. Moreover, TSC2 and Raptor (mTOR-associated regulatory protein) can be phosphorylated by AMPK, so as to make mTORC1 inactivate, and affect the activity of VPS34 by phosphorylating the serine 93 and 96 sites of the autophagy gene Beclin1, so as to induce autophagy (100, 101). Notably, under the condition of glucose starvation, AMPK will be activated, leading to the phosphorylation of threonine 388 site of the autophagy gene Beclin1, prompting the dissociation of the autophagy gene Beclin1 from Bcl2, and promoting the binding of Beclin1 to VPS34 and Atg14L. This change in dissociation and binding leads to the extremely strong catalytic activity of VPS34, which can generate a large amount of PtdIns3P, and promotes the rapid formation of autophagic vesicles, so as to greatly promote the occurrence and development of autophagy (102, 103).

Multitudinous studies have shown that AMPK/mTOR pathway can regulate the occurrence and development of atherosclerosis, myocardial hypertrophy, myocardial I/R and other diseases. Atherosclerosis is a chronic inflammatory process of aorta and middle artery wall, which starts from the interaction of wall cells, lipoproteins and inflammatory cells, and ends with the formation of plaque. AMPK can not only regulate the proliferation, migration, apoptosis and autophagy of vascular endothelium and smooth muscle cells, but also affect the biological function of macrophages, which plays a central role in the formation of atherosclerosis. AMPK is also involved in the metabolism of sugar, lipid and protein in the whole body, and then affects the level of blood sugar and lipid. The wall cells, especially vascular smooth muscle cells, play an important role in the progression of atherosclerosis. Studies have shown that AICAR can inhibit the proliferation of human aortic smooth muscle cells. After the activation of AMPK, cells in G0/G1 phase are increased, and cells in S phase and G2 phase are decreased, suggesting that AMPK can cause cell cycle arrest. AMPK can also regulate the antioxidation of vascular endothelial cells, decreasing the activity of AMPK can increase ER stress, which is conducive to the formation of atherosclerosis (104). Recent research have shown that AMPKα2 plays a major role in the inhibiting endothelial ER stress (105). In addition, AMPK can inhibit the proliferation of macrophages induced by oxidized low density lipoprotein (106). Hence, AMPK may be a novel therapeutic target of anti-atherosclerosis.

Other studies have shown that aortic ligation caused rats with myocardial hypertrophy are increased the activity of AMPK in the myocardium. Pharmacological activation of AMPK can reduce myocardial hypertrophy related to the overload of hemodynamic pressure. The activation of AMPK inhibits translation and transcription in the process of protein synthesis, directly phosphorylates eucaryotic elongation factor 2 kinase (eEF2K) to inhibit protein prolongation, inhibits mTOR signaling pathway, so as to inhibit the process of cardiac hypertrophy. LKB1 is absented in myocardium, the subunit of AMPK-α2 is Restricted activation, increases the activity of mTOR signaling pathway, leading to energetic shortage, and increases the expression of VEGF, so as to impair cardiac function (107). Studies have shown that the decrease of AMPK is accompanied by abnormal cardiac function, while metformin can protect cardiac function (108). Ang II is an important factor in cardiac hypertrophy and myocardial fibrosis. AMPK can inhibit Ang II induced-cell proliferation through affecting the extracellular signaling regulatory system (109), suggesting that AMPK may be a drug target for treating heart failure, especially myocardial fibrosis.

The mechanism of AMPK to protect myocardial ischemia has become very clear, which is through the way of increasing glucose uptake and glycolysis. During the period of myocardial reperfusion, AMPK promotes fatty acid to oxide. The decomposition of fatty acid produces a large amount of ATP, but high levels of fatty acids are associated with reperfusion injury, and AMPK can promote fatty acid to oxide and reduce the efficiency of cardiac energy, suggesting that the increase of AMPK activity during reperfusion may be harmful (110).

Additionally, the latest research has found that Sesn2, as a highly conservative member of the stress inducing protein family, can not only be induced by upstream hypoxia, ER and oxidative stress, but also involves in the occurrence and development of cardiovascular diseases through downstream AMPK/mTORC1 signaling pathway mediated autophagy. Studies have shown that the knockout of Sesn2 can induce endothelial dysfunction through AMPK dependent pathway and accelerate the formation of LPS induced-myocardial fibrosis (111), while the over-expression of Sesn2 can activate AMPK to alleviate sepsis induced-myocardial dysfunction (112). In the umbilical vein endothelial cells, pigment epithelium derived factor (PEDF) induces the expression of p53 and Sesn2, and inhibits mTOR, so as to trigger autophagy (113, 114). Other studies have shown that over-expression of Sesn2 triggers mitochondrial autophagy, enhances mitochondrial function, and improves myocardial dysfunction induced by doxorubicin (115). Moreover, over-expression of Sesn2 can reverse the autophagic signal inhibited by hyperlipidemia through AMPK/mTORC1 pathway (116). Therefore, Sesn2 can activate downstream AMPK and inhibit mTOR signal to induce autophagy, so as to regulate the occurrence and development of cardiovascular diseases, suggesting that Sesn2 may be a pharmacological target for treating cardiovascular diseases.

### MAPKs signaling pathway (ERK, JNK, p38)

MAPKs signaling pathways are existed in most cells. When extracellular stimulus signals are transferred to cells and their nuclei, they will trigger multifarious biological reactions (including cell proliferation, differentiation, transformation and apoptosis). Studies have shown that MAPKs signaling pathways are highly conservative in biological evolution of cells, and multiple parallel MAPKs signaling pathways have been found in both low-grade primary cells and high-grade mammalian cells. In addition, different extracellular stimuli can affect different MAPKs signaling pathways and mediate different biological responses through their mutual regulation. Mammalian MAP kinases can be divided into three categories according to their structure and function: (1) Stress-activated protein kinases (SAPKs) or c-Junn N-terminal kinases (JNKs). (2) Extracellular regulatory kinases (ERKs). (3) p38 MAPK. Among them, ERKs signaling pathway mainly regulates cell differentiation and growth. JNKs and p38 MAPK signaling pathway play an important role in stress responses, such as inflammation, apoptosis and other (117). In recent years, more and more studies have found that MAPKs signaling family plays an important role in the regulation of autophagy (118–120). For example, p38 MAPK can promote or inhibit autophagy under different cell types and cellular environments (121, 122). Therefore, in view of the double-sided role of autophagy in cardiovascular diseases, this pathway can be explored as a focus.

### P53 signaling pathway

p53 is not only an important gene of inhibiting tumor, but also closely associated with apoptosis. It can be subdivided into two types: mutant type and wild type (123, 124). Mutant-type p53 gene is mainly used to promote cell growth and participate in tumor occurrence. The major function of wild-type p53 gene is to involve in the negative modulation of cell growth and the expression of apoptotic regulation (125–127). Further studies have shown that wild-type p53 gene can promote the release of cytochrome C and other apoptosis inducing factors by affecting cell cycle and the change of mitochondrial Bcl-2/Bax ratio, start Caspase protease-cascaded reaction and activate the occurrence of myocardial cell apoptosis, so as to promote the apoptosis of cardiovascular system, and play an omportant role in the pathogenesis of myocardial infarction, heart failure, atherosclerosis and other cardiovascular diseases (128). Currentlly, different types of p53 genes are used as targets for the therapeutic strategy of cardiovascular diseases (129). Many discoveries also have indicated that autophagy inhibits p53 and p53 activates autophagy (130).

On the one hand, when myocardial cells express completely functional wild-type p53, they can recover from physiological apoptosis, while inhibiting wild-type p53 can delay the progress of cardiovascular diseases. MiR-146a attenuates apoptosis and regulates the role of autophagy in adriamycin induced cardiotoxicity through targeting TAF9b/p53 pathway (131). Polygonatum polygonatum polysaccharide, which is a polysaccharide compound extracted from polygonatum polygonatum, plays an anti-oxidant role in the mole of d-galactose induced-heart failure through ROS/p53 signaling pathway (132). Therefore, these evidences indicate that down-regulation of wild-type p53 has a potential protective effect on cardiac structure.

On the other hand, some mutational p53 genes have a novel function of promoting cell growth, called “function enhancement”, which is different from the original function and function of wild-type p53. Therefore, activation of these mutated p53 genes may be effective in the treatment of cardiovascular diseases. PRIMA-1 repairs and stabilizes the pre-existing p53 DNA binding domain and p53 repeat mutants in HCC cell lines (133). RETRA promotes the release of TAP73 by disturbing the TAP73/p53 mutant complex and enhancing its expression. Subsequently, the released p73 is participated in many processes of cell cycle arrest and apoptosis *via* activating p53 target gene (134). Hence, these mutant p53 genes are very valuable as potential targets for the growth of cardiovascular related cells.

### Nrf2/p62 signaling pathway

Nrf2/ARE pathway plays a wide range of protective roles in anti tumor, anti apoptosis, anti stress, nervous system and so on. Meanwhile, Nrf2 can play a pivotal role in regulating the stability of cardiovascular system *via* inhibiting oxidative stress. Therefore, Nrf2 is a potential target for the treatment of cardiovascular diseases. Studies have shown that laminar flow can promote Nrf2/ARE signaling transmission in human aortic endothelial cells, inhibit cytotoxicity induced by oxidative stress, inhibit the expression of monocyte chemoattractant protein-1 (MCP-1) and vascular cell adhesion molecule-1 (VCAM-1) induced by tumor necrosis factor α (TNF-α), also inhibit the adhesion of monocytes (135, 136). In addition, preventing the arteriosclerotic bloodstream can promote the expression of anti-inflammatory factor KLF2, which can enhance the anti-oxidative activity of Nrf2 (137). Other studies have shown that in vascular smooth muscle cells, oxidative stress can lead to nuclear translocation of Nrf2, so as cause the expression of Nrf2/ARE responsive genes (including A170, HO-1 and Prx1) (138). In the process of oxidative stress or ER stress, the activation of Nrf2/HO-1 pathway contributes to the survival of vascular smooth muscle cells, while the activation of Nrf2 can inhibit the proliferation of vascular smooth muscle cells (139). Notably, the growth of vascular smooth muscle cells can be blocked by NO dependent oxidative reactant (nitrolinoleic acid), and Nrf2 plays an important role in controlling its function (140).

There is evidence to prove that Nrf2/ARE pathway also plays an important role in the pathogenesis of cardiac disease, and several target genes of Nrf2 (including HO-1 (141), SOD (142), Trx (143) and GPx (144) play a key role in protecting cardiac remodeling and cardiac dysfunction. The loss of Nrf2 can increase the susceptibility of cardiac fibroblasts and myocardial cells to ROS and ROS induced cytotoxicity (145). Moreover, Nrf2 plays a key role in resisting oxidative injury of myocardial cells induced by high glucose (146). Other studies have shown that the expression of Nrf2 is up-regulated at the early stage of myocardial remodeling, but with the further decline of cardiac function, the expression of Nrf2 is decreased, and accompanied by cardiac dysfunction and the overload of pathological pressure. Subsequently, adenovirus over-expression of Nrf2 inhibits the hypertrophy of myocardial cells and the proliferation of cardiac fibroblasts. In vitro experiments, over-expression of Nrf2 inhibits oxidative stress of myocardial cells, while knockdown of Nrf2 expression has the opposite effect. Moreover, under the stressful state of pathological hemodynamics, the deletion of Nrf2 blocks the up-regulation of its target genes (including HO-1, NQO1, SOD2, SOD3, Trx1, Trx1R and Gpx), so as to cause abnormal myocardial remodeling, including increase of oxidative stress, apoptosis or fibrosis of myocardial cell, myocardial hypertrophy and early manifestations of cardiac dysfunction (135). Therefore, Nrf2 maintains the homeostasis of redox *via* regulating the expression of anti-oxidative genes and is a novel negative regulator of pathological myocardial hypertrophy and heart failure.

The phosphorylation of p62/Sqstm1 at Ser349 site enhances the tolerance and proliferation of cells to drugtoxicity *via* activating the transcribed factor Nrf2. Further studies have shown that hesperidin activates the activation of phosphorylated-p62 dependent Nrf2, which can protect the heart of mouse from arsenic trioxide damage (147). Diosmetin plays a protective role in myocardial hypertrophy *via* activating the p62/Keap1/Nrf2 signaling pathway dependent on phosphorylated-p62 (148). Hesperidin, diosmetin and other drugs may be used as exploratory drugs to treat cardiovascular diseases in the phosphorylated way of p62. Other studies have shown that Tsg101 positively regulates the p62/Keap1/Nrf2 pathway to protect the heart from oxidative damage (149). Therefore, Tsg101 may be a targeted drug for the treatment of cardiovascular diseases through the p62/Keap1/Nrf2 pathway. p62 is also an important autophagy marker, which can indicate protein accumulation and degradation. By interacting with Keap1/Nrf2, which plays an important role in regulating oxidative stress, while p62 is participated in cell survival, growth and death in cardiovascular diseases (150, 151). At present, p62/Keap1/Nrf2 targeting system and its related pathways (such as autophagy) are potential strategies for the treatment of cardiovascular diseases. Recent studies have discussed and reviewed the more detailed molecular mechanisms of cardiovascular disease therapeutic perspective in term of targeting Nrf2/p62 through autophagy (152, 153).

### Wnt/β-catenin signaling pathway

Wnt is a kind of secreted glycoprotein, which play a role through autocrine or paracrine. Postsecretory Wint can interact with specific receptors on the cellular surface and cause the accumlation of β- catenin. β- catenin, as a multifunctional protein, can interact with E-cadherin at the cellular junction and involves in the formation of adhesive bands, while free β-catenin can enter the nucleus and regulate the expression of related gene. Its abnormal expression or activation can cause cardiovascular diseases. The main components of Wnt signaling pathway include: (1) Wnt/β-catenin signaling pathway is used to activate gene and transcribe. (2) Wnt/PCP signaling pathway mainly regulates cytoskeletal rearrangement through small G protein-activated JNK. (3) Wnt/Ca^2+^ signaling pathway mainly affects cellular adhesion and the expression of related gene *via* releasing intracellular Ca^2+^. Among them, the typical regulation of Wnt pathway is induced by the binding of Wnt protein to Frizzled family cell surface receptors, leading to the activity of downstream effector Disheveled, which blocks the phosphorylation of β-catenin and promotes the translocation from cytoplasm to nucleus (154).

Recent research have found that Wnt/β-catenin signaling pathway is participatd in the process of atherosclerosis. Gelfand et al. (155) have found that the mouse endothelium of aorta appears atherosclerosis, the level of β-catenin increases significantly in the vulnerable area, while the β-catenin is blocked outside the nucleus in the protected area. In late plaque, intranuclear β-catenin also increases, and the environment of vulnerable area can activate the β-catenin/TCF signaling pathway. Sumida et al. (156) have found that hypertension changes the structure of vascular, leading to coronary atherosclerosis and irreversible damage of target organ, while Wnt1/β-catenin pathway may be involved in this process. C1qa gene knockout can inhibit the activity of this pathway, reduce the proliferation and fibrosis of vascular smooth muscle cells, improve pathological vascular remodeling and reduce the occurrence of atherosclerosis.

Heart failure is the end period of a varity of cardiovascular diseases developing into cardiac insufficiency. In the acute ischemic heart injury, Wnt1 is firstly up-regulated in epicardium, and then expresses on cardiac fibroblasts in the damaged area. Wnt1 can induce the proliferation and expression of cardiac fibroblasts to promote myocardial fibrosis, so as to cause cardiac injury and decrease cardiac function. Inhibition of Wnt pathway can significantly improve cardiac function (157, 158). Bastakoty et al. (159) established a rat model of myocardial infarction, and intravenously injected pharmacological inhibitor (gnf-6231) to inhibit the activity of Wnt. They found that compared with the control group, Wnt inhibition group enhanced the proliferation of myocardial cells, inhibited myocardial cells apoptosis, reduced the proliferation of myofibroblast around the infarct area and the synthesis of myocardial fibroblast collagen, and reduced adverse remodeling and the area of myocardial infarction. This study has shown that Wnt/β-catenin signaling pathway may play an important role in heart failure after myocardial infarction. In mice undergoing coarctation of the aorta, the cardiac pressure was overloaded, so as to increase the activation of Wnt/β-catenin signaling pathway and degree of cardiac fibrosis. This study indicates that losing the function of β-catenin can significantly improve cardiac function and reduce the occurrence of heart failure (160).

The role of Wnt/β-catenin pathway in myocardial injury has been paid more and more attention and the related molecules of Wnt classical pathway have increased the expression in myocardial infarction. Early research have found that the Dvl gene in Wnt/β-catenin pathway can be detected from the infarct border area 1 day after myocardial infarction in rats, and its expression increases 4 days later, reaching the peak at 7 days, which proves that the Dvl gene may be related to the proliferation and migration of myofibroblasts and vascular endothelial cells (161). Researchers use transgenic mice of LEF/TCF/β-catenin with β-galactosidase gene to establish a model of acute myocardial infarction. The positive emission tomography (PET) have shown that the metabolic activity of bone marrow is significantly increased. In addition, the level expression of Axin-2 in the β-catenin signaling pathway is also increased 7 days after acute myocardial infarction (162). Malekar et al. (163) have found that Wnt/β-catenin pathway plays an important role in the ventricular remodeling and the proliferation of TGF-β-iduced cardiac fibroblasts, so as to cause myocardial fibrosis and the decrease of cardiac function.

### NF-κB signaling pathway

NF-κB can regulate multiple biological functions, in addition to being indispensable in immune response and inflammatory response (164), the regulatory transcriptional program of NF-κB is extremely important for the maturation of the immune system, and the normal development of skeletal system and epithelial cells (165, 166). Its abnormal activation can lead to serious consequences such as cancer, autoimmune disease, neurodegeneration, cardiovascular disease, diabetes and so on (167). The biological pleiotropy of NF-κB is reflected in three aspects: (1) Involving pro-inflammatory reactions, NF-κB is the first line of defense to against infectious diseases, and also involves in cellular stress, immune response, and acute inflammatory response. (2) Anti-apoptosis, NF-κB induces the transcription of several antiapoptosis-related proteins, such as Bcl XL, TNF-related receptor factor, c-inhibitor apoptosis protein 1 (c-IAP1), c-inhibitor apoptosis protein 2 (c-IAP2) and so on. NF-κB can achieve the effect of antiapoptosis through controlling the core process of apoptotic process, which is the activity of apoptotic protease. (3) Promoting cellular growth, activated NF-κB can enhance the expression of cyclin-D1, so as to effectively control the process of cell cycle (168).

The NF-κB pathway can involve in double-sided regulation of various pathological processes of atherosclerosis. At the early period, oxidized lipoproteins infiltrates into the vascular intima and appears the expression of endothelial chemokines and adhesion factors, which lead to the migration of monocytes. At this period, NF-κB pathway regulates the expression of cyclooxygenase, lipoxygenase, cytokine, chemokine and sequestration factor (169). Following with the development of disease, NF-κB pathway regulates the gene expression of macrophage colony stimulating factor (M-CSF), stimulates the differentiation of infiltrating macrophages and transforms them into foam cells. NF-κB also regulates cellular factors, including interleukin-1 β (IL-1 β), TNF-α, interleukin 6 (IL-6), interleukin 12 (IL-12), Interferon γ (IFN γ), and indirectly involves in the low-grade inflammatory reaction in atherosclerosis. Interestingly, interleukin 10 (IL-10), as a recognized anti-atherosclerotic factor, is also affected by the regulation of NF-κB pathway (170). Hence, the role of NF-κB in atherosclerosis is not unidirectional. In addition, in the process of plaque development and rupture, the degradation of intracellular matrix is an important link, while NF-κB pathway can regulate the process of decomposing vascular basement membrane and rupturing plaque *via* the matrix metalloenzymes MMP-2 and MMP-9. Meanwhile, NF-κB is also regulated by other cytokine metabolites or pathogens, including interleukin 18 (IL-18), ox-LDL, advanced glycation end (AGE) products, cytomegalovirus (CMV) antigen, chlamydia, platelet derived factor, CD40L and so on (171). Moreover, the activation of NF-κB is also positively correlated with the value of C-reactive protein (CRP). And there is a evidence to prove that the activation of NF-κB in acute coronary syndrome may cause CRP to expand the scope of inflammation and affect the clinical effect (172).

During the myocardial I/R injury, the protective effect of NF-κB signaling pathway on the heart is beyond doubt. Research has shown that Bcl-2 can inhibit myocardial cells apoptosis, and its mechanism involves the IKK β-mediated NF-κB activity. And in the cultured myocardial cells *in vitro*, the over-expression of non-phosphorylated IKK β can cause the deactivation of NF-κB signal, so as to improve the TNF-α-induced apoptotic sensitivity (173). Studies have also shown that the specific expression of IKK β-mutated myocardium can increase the apoptosis of human myocardial cells, so as to increase the degree of acute coronary artery occlusion (174). Moreover, the p50 knockout rats increase the severity of cardiac insufficiency, leading to an increase in the incidence of myocardial infarction (175). Notably, the polymorphism of human p50 gene is also associated with the functional deterioration in patients with heart failure (176, 177). In addition, studies have also reported that the over-expression of IKK β can reverse hypoxia-induced apoptosis and mitochondrial defects (178). Hypoxia increases the expression of cell cycle transcription factor E2F-1, and the activated E2F-1 binds to the proximal promoter region of Brip3 to induce the expression of Brip3, so as to promote cell death. IKK β can interact with HDAC1 physically, inhibit the expression of Brip3 in Bcl-2 family members, and promote cell survival in hypoxic environment (179). Moreover, p65 can also compete with E2F-1 by adjacent κB site and bind to the region of Brip3 promoter region, so as to inhibit E2F-1-induced cardiomyocyte apoptosis and enhance the ability of cells to withstand hypoxia (180).

## Core molecular mechanism targeted therapy for cardiovascular disease

Because autophagy core mechanism plays an important role in regulating the process of cardiovascular diseases, pharmacological research and development of cardiovascular drugs based on autophagy targets have gradually become a investigative hotspot. Some studies have found that carvedilol can significantly promote the formation of autophagic vesicles in cells after acute myocardial infarction, and promote the expression of autophagy-related proteins and antiapoptosis-related proteins in the infarcted border area and infarcted area (181). The non-lipid regulating effect of statins has become a investigative hotspot in recent years. Some studies have shown that atorvastatin can alleviate left ventricular function and remodeling in rat model of spontaneous hypertension, which may be achieved by regulating autophagy of myocardial cell through Akt/mTOR pathway (182). Melanotropin is a hormone secreted by the middle lobe of pituitary gland. It has been found that in the rat model of ischemic heart failure, it can activate autophagy through cAMP and MAPK/ERK1/2 pathways, so as to prevent myocardial ischemic injury and delay the process of myocardial remodeling (183). Autophagy is not only participated in the pharmacological effects of cardiovascular drugs, but also closely associated with the adverse cardiac reactions of chemotherapeutic drugs. Currently, nthracyclic drugs are the most commonly used chemotherapeutic drugs in the field of tumor therapy. However, myocardial toxicity is also its largest adverse reaction, which is related to interfering autophagy. Doxorubicin inhibits the expression of transcription factor EB, so as to reduce the expression of autophagic protein, inhibit autophagic flow, make myocardial cells vulnerable to the toxicity and damage of DOX protein, and increase the susceptibility of DCM (184). Daunorubicin also has adverse effects of myocardial toxicity, and is also associated with significantly up-regulating the expression of autophagic marker protein Beclin-1 and LC3 (185). It can be seen that various cardiovascular active drugs have regulatory effects on autophagy. They can intervene cardiovascular diseases by regulating autophagy, and can also excessively promote autophagy, leading to myocardial injury.

Following with the deepening research of autophagic mechanism, the study on Chinese medicines and autophagic regulation has gradually attracted the attention of researchers, including curcumin, polydatin, luteolin, berberine, danshensu and so on. Han J et al. (186) have found that when human umbilical vein endothelial cells are subjected to oxidative stress, curcumin can induce autophagy through FOXO1 signaling pathway and protect vascular endothelial cells from oxidative stress. In the mouse model of acute myocardial infarction, polydatin can activate Sirt3, up-regulate autophagic flux of myocardial cells and improve mitochondrial dysfunction, so as to protect myocardial cells from myocardial infarction injury (187). Hu J et al. (188) have found that in the model of myocardial infarction, luteolin can up-regulate autophagic flux of myocardial cells and improve mitochondrial viability through Mst1 inhibitor, so as to alleviate myocardial dysfunction. Contrary to the cardiovascular protection by inducing autophagy, some Chinese medicines can also inhibit the excessive expression of autophagy. Huang Z et al. (189) have found that berberine can alleviate myocardial IRI by inhibiting the over-expression of autophagy-related proteins (SIRT1, BNIP3 and Beclin-1). Fan et al. (190) have found that danshensu can inhibit IRI-induced excessive autophagy through activating mTOR signaling pathway and reduce cell apoptosis, so as to alleviate myocardial cells IRI and improve cardiac function. It can be seen that Chinese medicines have a double-sided regulation on autophagy, which can not only promote autophagy to reduce adverse cardiovascular events, but also inhibit excessive autophagy to improve the survival rate of myocardial cells. At present, many compounds are extracted from traditional Chinese medicines, and used *in vivo* or *in vitro* and clinical trials for the treatment of cardiovascular diseases. Here, we summarize these compounds to clarify that Chinese medicines affect the occurrence and development of cardiovascular diseases through bidirectional regulation of autophagic targets (Table 2).

**Table 2:** Chinese medicines mediated-autophagy has doule-sided regulation in cardiovascular diseases.

Compound	Chinese medicine	Signaling pathway or target	Effects	References
Sinomenine	Caulis sinomenii	ERK/mTOR, HMGB1	Induction of autophagy resists inflammation in EA.hy926 cells	(191)
Seabuckthorn flavone	Hippophae rhamnoides Linn.	LC3-I/II, Beclin-1, SIRT1, NADPH	Increasing the level of autophagy reduces the degree of atherosclerosis	(192)
Salidroside	Rhodiola rosea L.	PI3K/Akt/Nrf2, AMPK/mTOR	Induction of autophagy protects myocardia cells from the hyperglycemic or hypoxia injury	(193, 194)
Resveratrol	Veratrum nigrum L.	AMPK/mTOR, p38, LC3-II, Beclin-1	Induction of autophagy protects H9C2 embryonic rat heart derived cells from oxidative stress	(195)
Polydatin	Reynoutria japonica Houtt.	Nfr2/HO-1, SIRT3	Increasing the level of autophagy protects cardiomyocytes against myocardial infarction injury	(187, 196)
Luteolin	Reseda odorata L.	MST1, p62, LC3-1/II, Beclin-1	Induction of autophagy enhances the viativity of myocardia cells	(188)
Curcumin	Curcuma longa L.	FOXO1	Induction of autophagy protects vascular endothelial cell survival from oxidative stress damage	(186)
Astragaloside IV	Astragalus membranaceus (Fisch.) Bunge.	PI3K/Akt/mTOR, LC3-I/II	Protective effect of mediated autophagy on hyperglycemic injury of H9C2 (2-1) myocardial cells	(197)
Hesperidin	Citrus reticulata Blanco.	PI3K/Akt/mTOR, LC3-II, Beclin-1	Inhibition of autophagy contributes to resist myocardial ischemia/reperfusion injury	(198)
Diosmetin	Dioscorea oppositifolia L.	p62/Keap1/Nrf2, LC3I/II, Beclin-1, ATG7	Inhibition of autophagy plays a protective role in myocardial hypertrophy	(148)
Danshensu	Salvia miltiorrhiza Bunge	mTOR signaling, p62, p-S6, p-S6K1	Inhibition of autophagy alleviates cardiac ischaemia/reperfusion injury	(190)
Salvianolic acid B	Salvia miltiorrhiza Bunge	NIX, LC3-I/II	Inhibition of mitochondrial autophagy protects H9C2 cells from the hypoxia/reoxygenation injury	(199)
Allicin	Garlic	PI3K/Akt/mTOR, ERK/mTOR	Inhibition of autophagy reduces pathological myocardial hypertrophy	(120)
Ginsenoside Rb1	Panax ginseng C. A. Meyer	NF-κB, LC3-II, p62	Inhibition of autophagy protects the adriamycin-induced injury in myocardia cells	(200, 201)
Berberine	Coptis chinensis Franch.	AMPK/mTOR, SIRT1, BNIP3, Beclin-1	Inhibition of the excessive autophagy alleviates cardiac ischemia/reperfusion injury	(189)

## Signaling cascades targeted therapy for cardiovascular disease

PI3K/Akt/mTOR pathway plays an important role in the occurrence, development and treatment of cardiovascular diseases by regulating autophagy. Due to the bidirectional role of autophagy in cardiovascular diseases, the importance of balancing autophagic activity of PI3K/Akt/mTOR pathway in the treatment of cardiovascular diseases becomes very important. Studies have shown that the abnormal activation of Akt and PI3K in cardiomyocytes leads to the activation of PI3K/Akt/mTOR pathway, which promotes the proliferation of myocardial cells by the activating proteins synthesis *via* p70S6K and 4EBP1 phosphorylation. Meanwhile, inhibiting PI3K/Akt/mTOR pathway can also activate autophagy and enhance the survival of myocardial cells under oxidative stress (202). These evidences suggest that activation or inhibition of PI3K/Akt/mTOR pathway may be beneficial to the treatment of cardiovascular diseases. On the other hand, the activation of autophagy does not inhibit the progression of cardiovascular diseases in the later period. In the later period of cardiovascular diseases, excessive activation of autophagy *via* inhibition of PI3K/Akt/mTOR pathway may cause autophagic cell death, so as to exacerbate the development of cardiovascular diseases (203). Hence, in view of the controversial situation of PI3K/Akt/mTOR in cardiovascular diseases, we can carefully consider the different period of cardiovascular diseases and the control of autophagic activity.

Excessive activation of AMPK/mTOR pathway induces autophagy, promotes autophagic death and inhibits cardiomyocyte growth (204, 205). Activation of autophagy can also help cells survive under stressful conditions, such as hypoxia, chemotherapy, starvation and so on (206). In addition, it is reported that inhibiting autophagy may promote cell death by increasing the sensitivity of myocardial cells to cytotoxic drugs (207). In conclusion, it is necessary to further emphasize the bidirectional role of AMPK/mTOR pathway in cardiovascular diseases.

So far, the researches on the modulative role of Ras/Raf/MEK/ERK pathway in cardiovascular diseases through autophagy are still finite. Lately, evidence has revealed that Ras/Raf/MEK/ERK pathway *via* regulation of autophagy by mediating mTOR plays an important role in cardiovascular diseases (208). Notably, mTOR is a major regulator participated in PI3K/Akt, AMPK and Ras/Raf/MEK/ERK pathways. These three mTOR-mediated pathways show multiple intersections rather than independent parallel pathways (209).

Mutational p53 is most commonly appeared in human cancer and can also regulate apoptosis. Moreover, different types of p53 have dual functions of regulating autophagy in cardiovascular diseases. On the one hand, when cells are under stressful conditions, wild type nuclear p53 can be used as a transcribed factor to induce autophagic genes. On the other hand, cytoplasmic p53 inhibits autophagy. Compared with the nuclear p53, cytoplasmic p53 inhibits AMPK, which a positive regulator of autophagy, and further activates mTOR to inhibit autophagy. Notably, in the absence of cytoplasmic p53, excessive activation of wild type nuclear p53 can induce autophagy and cause autophagic cell death (210, 211). Therefore, the mutational status of myocardial cells and the crosstalk of different types of p53 paricipated in regulating autophagy and apoptosis need to explore deeply.

NF-κB pathway in immune response and inflammatory response are indispensable (164). The transcriptional programs regulated by this pathway are extremely important for the maturation of the immune system and the normal development of the skeletal system and epithelial cells (165, 166). In addition, its abnormal activation can lead to serious consequences, including tumor, autoimmune disease, neurodegeneration, cardiovascular disease, diabetes and so on (167). However, there exists still a gap between the basic research and application of the chemical compound. The principle of the pharmacologic therapies design targeting NF-κB pathway need to consider the experimental model of different systems and environmental factors (212–214). The IKK-γ knockout mice can make NF-κB signal lose activity, so as to enhance TNF-α-mediated apoptotic sensitivity, while the excessive expression of phosphorylated IKK-γ can activate NF-κB signal and has a protective effect on the heart (173). Meanwhile, when myocardial cells are damaged by hypoxia, the excessive expression of IKK-β can reverse hypoxia-induced apoptosis and mitochondrial defects, so as to promote cell survival in hypoxic environment (178). Increasing evidences have shown that NF-κB also involves in regulating the occurrence and development of infection in cardiomyopathy (215). These researches indicate that compounds of targeting NF-κB pathway may be considered for the periods of cardiovascular diseases and the inflammatory condition.

p62, as a substrate for autophagy, is degraded during autophagic activation. At the same time, the accumulated p62 can also inhibit autophagy through activating mTORC1 (216). In addition, as we all known that inducing mTORC1 can promote the development of cardiovascular diseases. Hence, p62-mediated the activity of mTORC1 may also contribute to cardiovascular diseases. These evidences suggest that p62 may play the role of cardiovascular diseases promoter by regulating many signaling pathways, including mTOR and autophagy (217). Interestingly, due to the inhibition of autophagy by p62-mediated mTORC1 activation, we can consider combining autophagy-related inducers to control autophagic activity, which can not only enhance the tolerance of myocardial cells in stressful conditions, but also limit autophagic cell death caused by excessive autophagy. However, the rationality of autophagic activity also needs further consideration and verification.

Generally, either the induction or inhibition of autophagy may be beneficial to the treatment of cardiovascular diseases through targeting signaling pathways. At present, many drugs or chemicals have been used *in vivo* and *in vitro* for the treatment of cardiovascular diseases. Here, we summarize these compounds to clarify the double-sided role of autophagy inducers and inhibitors in cardiovascular diseases (Table 3).

**Table 3:** Targeting autophagy as a therapeutic strategy for cardiovascular diseases *via* signaling cascad.

Participated pathway	Drugs/Compounds	Induction (+)/Inhibition (−) of autophagy	Cardiovascular diseases	In vivo/vitro	References
PI3K/Akt/mTOR pathway	Mibefradil	Induction (+)	Cardiac hypertrophy	Vitro	(218)
	Ivabradine	Induction (+)	Myocardial infarction	Vivo	(219)
	Ginsenoside Rb1	Inhibition (−)	Myocardial I/R	Vitro	(220)
	Stem-leaf saponins	Inhibition (−)	Cardiac protection	Vivo	(221)
	Danlou tablets	Induction (+)	Coronary atherosclerosis	Vivo and vitro	(222)
IGF-1 and EGFR pathway	Spermidine	Induction (+)	Later period of cardiac disease	Vivo	(223)
	Morphine	Inhibition (−)	Myocardial I/R	Vivo	(224)
AMPK/mTOR pathway	Metformin	Induction (+)	Diabetic cardiomyopathy	Vivo and vitro	(225)
	Tanshinone IIA	Induction (+)	Heart failure	Vivo and vitro	(226)
	Chikusetsu saponin IVa	Induction (+)	Myocardial fibrosis	Vivo	(227)
	Nkx2-3	Induction (+)	Vascular remodeling disease	Vivo and vitro	(228)
	Melatonin	Inhibition (−)	Myocardial I/R	Vivo and vitro	(229)
MAPKs pathway (ERK/JNK/p38)	Vildagliptin	Induction (+)	Diabetic cardiomyopathy	Vivo	(230)
	Rab7	Induction (+)	Aortic dissection	Vitro	(231)
	Allicin	Inhibition (−)	Cardiac hypertrophy	Vivo and vitro	(120)
	DUSP1	Inhibition (−)	Myocardial I/R	Vivo and vitro	(232)
	Ghrelin	Inhibition (−)	Doxorubicin-induced cardiotoxicity	Vitro	(233)
	Cilostazol	Inhibition (−)	Cardiac hypertrophy	Vitro	(234)
P53 pathway	6-Bromoindirubin-3′-oxime	Induction (+)	Heart failure	Vivo and vitro	(235)
	17β-estradiol	Induction (+)	Coronary atherosclerosis	Vitro	(236)
	LncRNA CAIF	Inhibition (−)	Myocardial infarction	Vivo and vitro	(237)
Nrf2 pathway	Salidroside	Induction (+)	Diabetic cardiomyopathy	Vitro	(193)
	β-LAPachone	Induction (+)	Doxorubicin-induced cardiotoxicity	Vivo	(238)
	KLF2	Induction (+)	Coronary atherosclerosis	Vitro	(239)
	Lycium barbarum polysaccharide	Inhibition (−)	Myocardial I/R	Vivo and vitro	(240)
	Propofol	Inhibition (−)	Myocardial I/R	Vivo	(241)
Wnt/β-catenin pathway	Huoxin pill	Induction (+)	Myocardial ischaemia	Vivo and vitro	(242)
	SIRT1	Inhibition (−)	Coronary atherosclerosis	Vivo	(243)
NF-κB pathway	FK506	Induction (+)	Myocardial infarction	Vivo	(244)
	Aspirin	Induction (+)	Coronary atherosclerosis	Vitro	(245)
	MSTN	Inhibition (−)	Cardiac hypertrophy	Vivo and vitro	(246)

## Conclusions and future directions

**Figure 4:**
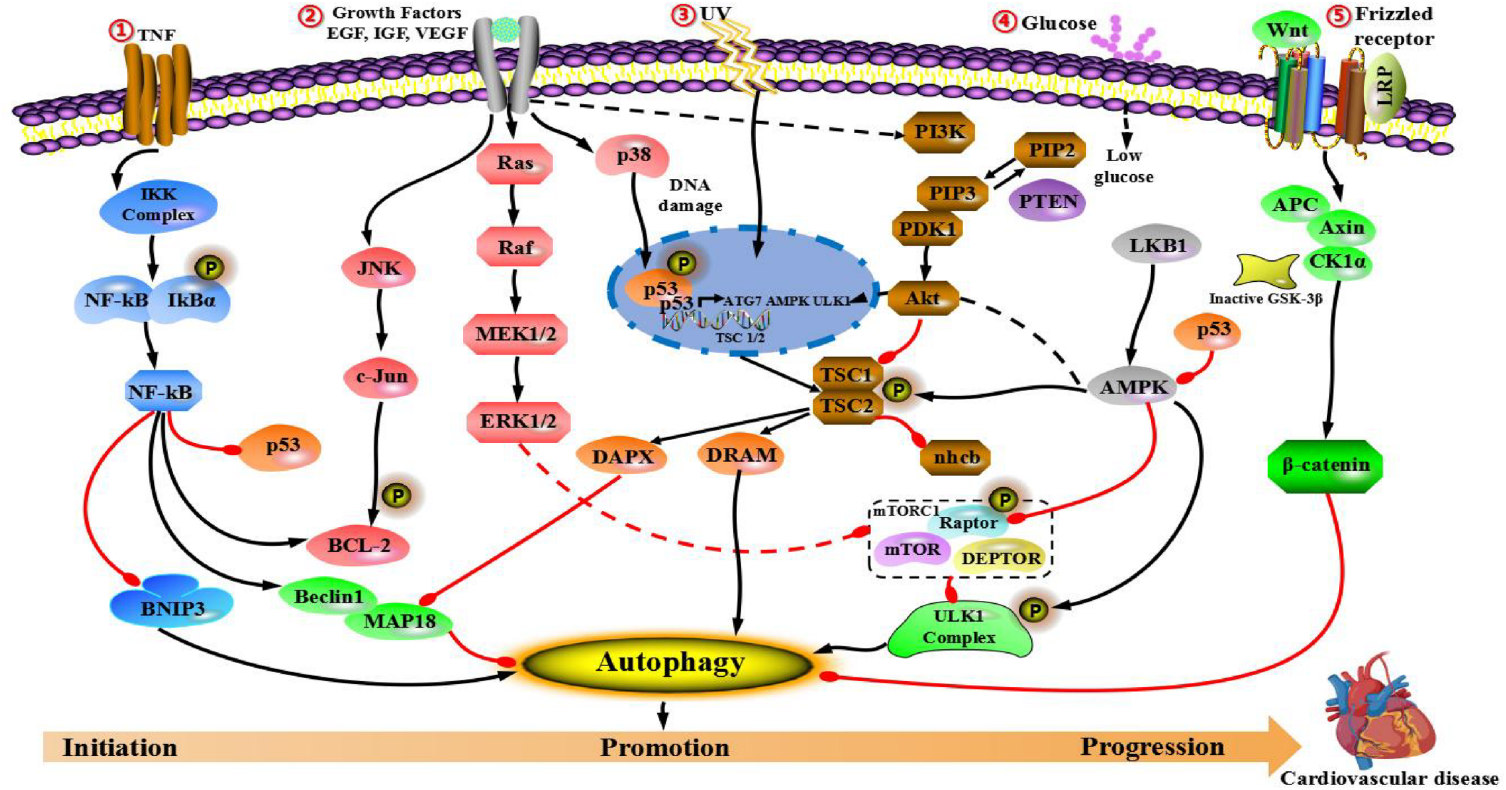
Regulation of cross- interference between autophagy and main signaling pathways in cardiovascular disease. (1) NF-κB pathway of IKKα/β and NF-κB induces autophagy by increasing the expression levels of autophagy-related proteins, such as Beclin-1. (2) Growth factors (including EGF, IGF and VEGF) promote autophagy by modulating the autophagic process of JNK/c-Jun, Ras/Raf/MEK/ERK and the PI3K/Akt. (3) p53 pathway: Autophagy is also mediated by the nuclear p53 activity, which is a transcribed factor under stressful conditions (such as ultraviolet rays, etc.). p53 induces the classic autophagic pathway mainly through PI3K/Akt/mTOR, AMPK/mTOR and ULK1 complex. (4) PI3K/Akt/mTOR and AMPK/mTOR receptor tyrosine kinases promote the transformation of PIP2 into PIP3 and the activation of PI3K. PTEN-induced dephosphorylation of PIP3 activates the AKT signal negatively regulated by PI3K. Amino acids and nutrient-rich conditions can initiate the activity of mTORC1 signal. By contrast, starvation and oxidative stress can inhibit the activity of mTORC1 signal and induce autophagy. (5) Wnt-β-catenin pathway: This pathway causes the activation and nuclear recruitment of β-catenin protein, so as to directly regulating autophagy.

Cardiovascular disease is a major cause of death that seriously endangers human health. The discovery of new risk factors, the establishment of innovative diagnostic methods, the improvement of treatment interventions and the improvement of doctors' and patients' cognition have significantly decreased the mortality of cardiovascular diseases at the period of appropriate age. However, following with the change of modern lifestyle and the prevalence of global obesity, the incidence of cardiovascular disease rises continuously (247). Hence, it is very necessary to identify and excavate new targets for the cardiovascular intervention. As outlined in this review, research on the development and treatment of cardiovascular disease can also focus on molecular and cellular signaling mechanisms. Autophagy is a highly selective and powerful process that is closely associated with the physiological functions of various cells (including myocardium, vascular endothelial cells and so on), so autophagy plays a crucial role in cardiovascular diseases, which may be regulated by several key signaling pathways (Figure 4). We hope that the key points for future cardiovascular therapy will be highly dependent on potential molecular pathways involved in autophagy. Our main task aims to construct a functional networks related to autophagy and cardiovascular treatment. In fact, some idea of the prospect for this mission can be given through the crosstalk of autophagy with many signaling pathways.

In this review, the following several issues are needed to be addressed in the future. (1) First of all, how to choose the diagnostic markers correctly before or during the treatment of cardiovascular disease? Due to the complexity of cardiovascular disease, we may also need to decide when and where to observe markers. In addition, the combination of several markers may make the results more reliable than using a single marker. (2) Secondly, when the use of different autophagy inducers (such as rapamycin and SMER18) or inhibitors (such as hloroquine diphosphate and 3-methyladenine) in combination with cardiovascular drugs or other small molecules, we need to elucidate the potential molecular mechanism, cytotoxin of these chemicals and the specific role of autophagy before proceeding to the next step. (3) Thirdly, these pathways are the most important signaling cascades and very complex molecular mechanisms, which are closely associated with the occurrence and development of cardiovascular disease. Moreover, autophagy can affect the development of heart and blood vessels at several levels in most pathways. These discoveries further confirm the multiplicity of autophagy in the occurrence of cardiovascular disease. Therefore, when and how to inhibit or activate the relevant pathways will provide the possibility for the cardiovascular treatment. (4) Eventually, finding specific inducers and inhibitors of autophagy is extremely important. Additionally, how to balance the mechanisms of autophagy-induced cell death and cell survival is a major concern in the treatment of cardiovascular disease. Another point of balance that we need to consider is how to determine the right period for the occurrence and development of cardiovascular diseases. Consequently, on the road of exploring the treatment of cardiovascular disease through autophagy, we should choose appropriate methods to obtain better efficacy, induction or inhibition of autophagy is entirely dependent on the specific cases and analyses.
